# Current Developments in Native Nanometric Discoidal Membrane Bilayer Formed by Amphipathic Polymers

**DOI:** 10.3390/nano11071771

**Published:** 2021-07-07

**Authors:** Mansoore Esmaili, Mohamed A. Eldeeb, Ali Akbar Moosavi-Movahedi

**Affiliations:** 1 Department of Biochemistry, University of Alberta, Edmonton, AB T6G 2H7, Canada; 2 Department of Neurology and Neurosurgery, Montreal Neurological Institute, McGill University, Montreal, QC H3A 2B4, Canada; eldeeb@ualberta.ca; 3 Department of Chemistry, Faculty of Science, Cairo University, Cairo 12613, Egypt; 4 Institute of Biochemistry and Biophysics (IBB), University of Tehran, Tehran 1417614411, Iran; moosavi@ut.ac.ir

**Keywords:** synthetic biology, heteropolymers, amphipathic, lipid bilayer, self-assembly, membrane proteins

## Abstract

Unlike cytosolic proteins, membrane proteins (MPs) are embedded within the plasma membrane and the lipid bilayer of intracellular organelles. MPs serve in various cellular processes and account for over 65% of the current drug targets. The development of membrane mimetic systems such as bicelles, short synthetic polymers or amphipols, and membrane scaffold proteins (MSP)-based nanodiscs has facilitated the accommodation of synthetic lipids to stabilize MPs, yet the preparation of these membrane mimetics remains detergent-dependent. Bio-inspired synthetic polymers present an invaluable tool for excision and liberation of superstructures of MPs and their surrounding annular lipid bilayer in the nanometric discoidal assemblies. In this article, we discuss the significance of self-assembling process in design of biomimetic systems, review development of multiple series of amphipathic polymers and the significance of these polymeric “belts” in biomedical research in particular in unraveling the structures, dynamics and functions of several high-value membrane protein targets.

## 1. Biopolymers, an Inspiration for Designing Smart Surface-Active Polymers

**Figure 1:**
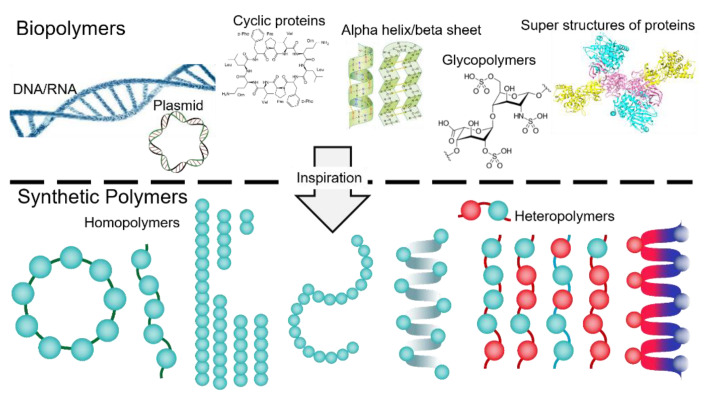
Schematic view of diverse biopolymers in cells, and their resemblance to chemically synthesized polymers that have been shown broad utilities in biomedical research. During the synthetic process of homo ad heteropolymers, optimizing the sequence, length and shape (linear vs. circular), backbone flexibility, stereochemistry, and homogeneity are crucially difficult to manage.

Within the cells, self-assembly is characterized as spontaneous organization of homo or hetero biomacromolucules (proteins, nucleic acids, lipids, carbohydrates) via non-covalent intrinsic forces (i.e., dipole-dipole interactions, π-π stacking, electrostatic forces, hydrogen bonding, metal–ligand interactions) which in turn results in fascinating supermolecular structures and machineries that govern the autonomous and self-sustaining functions of cells [1]. For years, the significance of biological self-assemblies was taken as granted, yet emerge of biophysical and imaging technologies as well as remarkable advancements in molecular biology opened novel perspectives on morphology, dynamics and functions of precise [2] and higher-ordered nanostructures in cell, and inspired ambitious efforts to design and synthesize similar artificial assemblies that display diverse innovative utilizations in biomedical research (Figure 1) [3,4].

**Figure 2:**
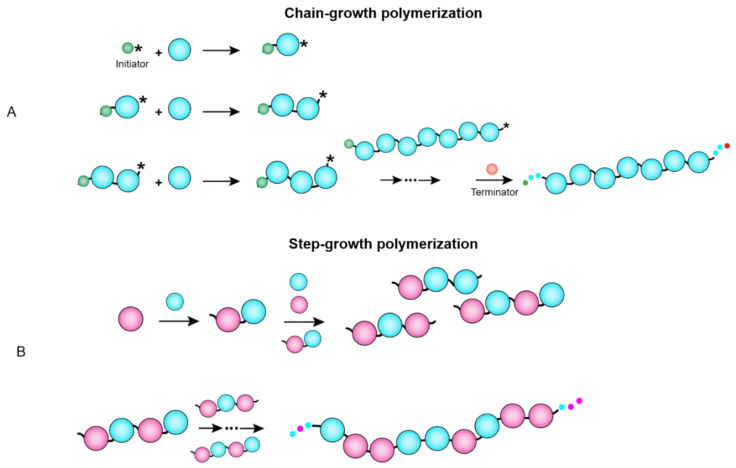
Random copolymerization of building blocks (cyan and pink circles) (**A**) via random chain growth and (**B**) step growth polymerization mechanisms. Asterisks represent the radical atoms. Initiator and terminator are, respectively, shown as in green and red.

Unlike proteins and nucleic acids, synthetic polymers are synthesized chemically by polymerization of synthetic co-monomers through either chain-growth or step-growth mechanisms. During chain-growth polymerization, an initiator triggers the formation of radical or ionized forms of each unsaturated monomer that then leads to chain propagation step in which monomers polymerize and repeatedly add to the length of polymers, finally upon addition of terminator, chain growth ends (Figure 2A). The step-growth mechanism, however, does not require any initiator or/and terminator since each monomer contains an active reaction site, and the condensation between monomers is followed by an elimination reaction in which another molecule, namely water, is released (Figure 2B) [5].

**Figure 3:**
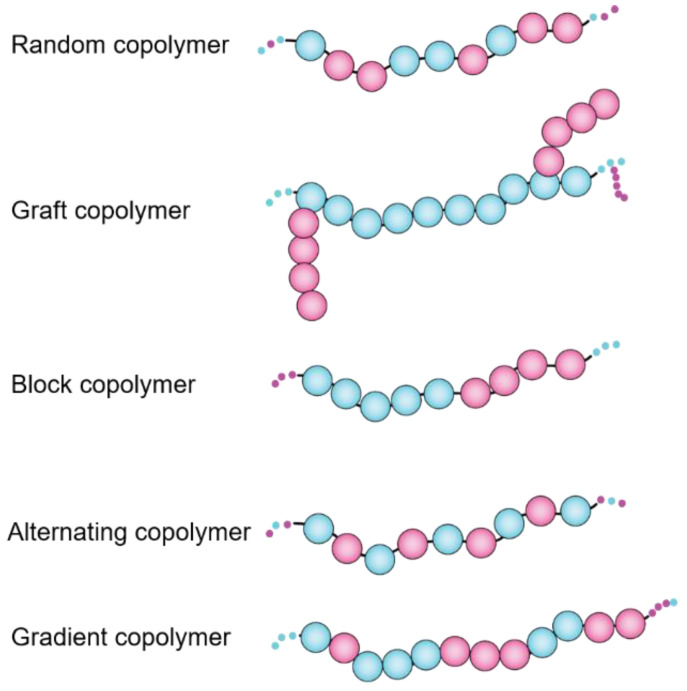
General arrangement of comonomers in heretopolymers lead to seven major classes of amphiphilic polymers.

Since the invention of synthetic rubber back in the 1930s, different sequence-customized categories of copolymers, including alternating, random, block, grafted, periodic, gradient, and copolymers have been evolved [6] (Figure 3), however none are barely comparable to biopolymers (DNA, protein, peptides) with precisely tunable sequences of comonomers. The physico-chemical characteristics of functional monomers, length of polymers, order (sequence) of comonomers along the polymer chains, as well as stereochemistry and flexibility of polymer backbone all contribute in the final behaviors of polymer molecules in solution and modulate their inter and intra molecular forces that affect rheology, morphology and surface-characteristics of polymers [7].

### Evolution of Amphipathic Polymers in Biological Systems

Possession of both lipophilic (apolar) and polar (hydrophilic) moieties in molecules leads to different degree of amphipathicity. Such molecules range from small molecule surfactants (i.e., chemical detergents) and biosurfactants (e.g., phospholipids). During evolution, mother Nature has optimized amphipathicity of biopolymers like proteins by incorporating variety of naturally available hydrophobic and hydrophilic building blocks (e.g., amino acids) tailoring their functions, and modulating their self-assembly and interactions with other components in the cells. Examples of such nanoscale multi-component assemblies include (1) membrane proteins machineries incorporated within phospholipid bilayers; (2) amphipathic apolipoprotein A–I (apoA1) as main constituents (~70%) of high-density lipoprotein (HLD) particles which involve a soluble, polydisperse population of lipid-protein complexes in the body responsible for transport of specific lipids such as cholesterol ester and other small molecule metabolites [8,9,10]; (3) mixed micelles of alpha and beta caseins in milk that incorporate with minerals and fat [11,12].

## 2. Natural Biopolymers as Tools for Spontaneous Reconstruction of Biomembrane Assemblies

### 2.1. Protein-Based Approach

The nanometric discoidal membrane bilayer, or nanodisc, was first replicated from full-length amphipathic apolipoprotein A–I (apoA1), the main constituent (~70%) of high-density lipoprotein (HLD) particles, which involve a soluble, polydisperse population of lipid-protein complexes in the body, responsible for transport of specific lipids such as cholesterol ester and other small molecule metabolites [8,9]. The engineering of apoA1 proteins made possible the production of a library of amphipathic protein “belts” of various sizes, which may be mixed with detergent-solubilized lipid-protein complexes [13,14,15,16]. In this process, upon removal of detergents from the mixture, the self-assembly process initiates, and protein-lipid natural tendency force them into highly-uniform, nano-sized lipid bilayer forms [17]. Finally, two copies of apoA1 proteins (helical membrane scaffold proteins (MSPs)) encircle the entire complex [18,19,20].

The availability of MSPs (some tagged with hexa/octa histidine or FLAG tags) in various sizes and the feasibility of manipulating the lipid-protein ratio enables scientists to design and build nanodiscs within a range of ~10–17 nm. Medium-sized MSP nanodiscs (formed of MSP1D1 and MSP1E3) [21] can accommodate 140–340 lipid molecules, while large ones (e.g., those composed of MSP2N2 with 16 helices) can accommodate up to ~650 lipids [22]. The thermal phase transition of the lipid bilayer differs slightly between small and large discs, affecting the respective lipid packing (elasticity/flexibility in lateral movements) and the possibility of expansion in discs of different sizes. However, small-angle X-ray scattering (SAXS) and differential scanning calorimetry (DSC) analyses suggest that the phase transition of lipids in MSP nanodiscs is significantly higher than that reported for multilamellar lipid vesicles (MLV) and unilamellar vesicles (liposomes) [20,23,24,25,26,27,28,29].

Unprecedented range of membrane proteins (some with up to 24 transmembrane helices) from various sources have been reconstituted into MSP discs, and both their conformational dynamics and interactions have been studied by cryo-electron microscopy (cryo-EM), solution-state nuclear magnetic resonance spectroscopy (NMR), and X-ray crystallography. MSP nanodiscs provide a superior system for in vitro reconstitution of membrane proteins to examine the role of lipid microdomain, and to observe the conformational changes of membrane proteins. Despite all the advantages of MSPs and their substantial impact on the field of membrane biology, this procedure is detergent-dependent and hence, natural lipids may be lost during the initial purification steps [18].

Linking the carboxylic and amine termini of engineered variants of apolipoprotein A1 (ApoA1) via covalent peptide bonds leads to novel circulated forms of membrane scaffold proteins that offer demonstrated advantages such as thermal stability and proteolytic resistance [30]. The circulation process occurs through several strategies including intein-fusion proteins, sortase transpeptidases, and chemical ligation. NW9, NW11, NW30, and NW50 are examples of nanoscale discs with approximately 8.5, 11, 15, 50 nm width, respectively.

### 2.2. DNA-Based Amphipathic Polymers

Nucleic acid-based polymers (NAP) such as REP 2055 and REP 2139 (composed of alternating adenosine and cytidine, respectively, and phosphodiester linkages) have shown to get engaged in hydrophobic interactions with exposed hydrophobic protein surfaces, blocking the viral life cycle before and after entry to the cells, hence promising anti-viral activity against hepatitis B and hepatitis D and HIV in a sequence-independent yet size-dependent manner [31,32].

Nanoscale 3D DNA objects (known as DNA origami) are another variant of bio-engineered systems with extensive applications in drug delivery, functional nanorobots and molecular motors, and basic research such as membrane biochemistry [33,34]. Such nano objects can be also used as scaffolding corrals for encapsulation of phospholipid bilayer in large discoidal DNA nandodisc with about 45 and 70 nm diameter, which can be also loaded with membrane proteins [35].

### 2.3. “Sweet” Sugar-Based Amphiphilic Polymers (SBAPs)

Naturally-occurring polysaccharides from different sources (e.g., starch from plant, dextran from bacteria, heparin from animal source), and chemically modified polymer scaffolds with glyco-conjugated moieties, and sugar-linked polymers constitute three major classes of SBAPs, and have received tremendous attention as nanocarriers for delivery of genes, drugs, and proteins as well as diagnostic devises [36,37]. SBAPs take various nano morphologies such as micelles, microgels, and nanoparticles [38] and are considered as less-immunogenic and easily tunable and biodegradable. Synthetic SBAPs are the results of either polymerization of glycosylated comonomers or various polymer backbone can be conjugated with variety of sugar molecules forming polymers with specific physico-chemical properties such as varying degree of charge (positive, negative or non-ionic) or hydrophobicity [39]. Chemically-modified inulin (high molecular weight fructo-oligosaccharides (FOS) or fructans) with pentyl, benzyl, hexyl, have been shown to effectively solubilized synthetic lipid bilayers composed of DMPC:DPMG (7:3) and form particles with ~10 nm hydrodynamic radius. These glycopolymers are remarkably tolerant to up to 100 mM divalent metal ions, and fully soluble pH range 2.5–8 [40].

## 3. Synthetic Polymers for Reconstitution of Membrane Assemblies

### 3.1. Short Synthetic Amphipathic Polymers (Amphipols)

Amphipols comprise hydrophobic (short alkyls C8–C10) and hydrophilic (charged groups, hydroxyl, glucose) moieties along the polymer chain and display a higher affinity for irreversibly interaction with transmembrane proteins than with small hydrophobic molecules such as lipids or ligands. Limited self-assembly (as defined by critical aggregation concentration) of amphipols into globular particles with a well-defined diameter may create a hydrophobic inner core suitable to incorporate membrane proteins. The hydrophobic core does not resemble the lipid bilayer; unlike detergent, it limits the release of lipids.

Amphipols are not adequately effective in liberating lipid-protein assemblies spontaneously. The preparation of the protein/amphipol complex is a multistep, detergent-dependent procedure in which detergent is exchanged out and desired lipids plus amphipols are added to the naked membrane protein (devoid from natural lipids), which may have undergone some conformation changes by this stage. However, amphipols offer advantageous thermal stability to proteins and, due to their low aggregation concentration, are cost-effective [41].

### 3.2. Long Amphipathic Polymers

Different classes of synthetic polymers been proven instrumental in membrane biology. The first example of such polymers are composed of styrene and maleic anhydride (MAn), the two hydrophobic building blocks of SMAn polymers, and the initial molar ratio of these two comonomers in polymerization batch determines the final ratio of styrene: maleic anhydride in final polymer chain. The anhydride form of SMA polymer (also known as XIRAN resin) (SMAn) is heat and chemical resistant plastic with wide application range for synthesis of automobile parts, plastic appliances, industrial dyes, and pigments. Historically, TOTAL Cray Valley (Exton, PA, USA) and Polyscope (Geleen, The Netherlands) were the two primary (yet not sole) suppliers of SMA copolymers, which respectively use SMA and SZ prefixes in their catalogs. Malvern Cosmeceuticals. Ltd. (Malvern Hills, UK) supplies SMA2000 under the commercial name of Lipodisq^®^.

**Figure 4:**
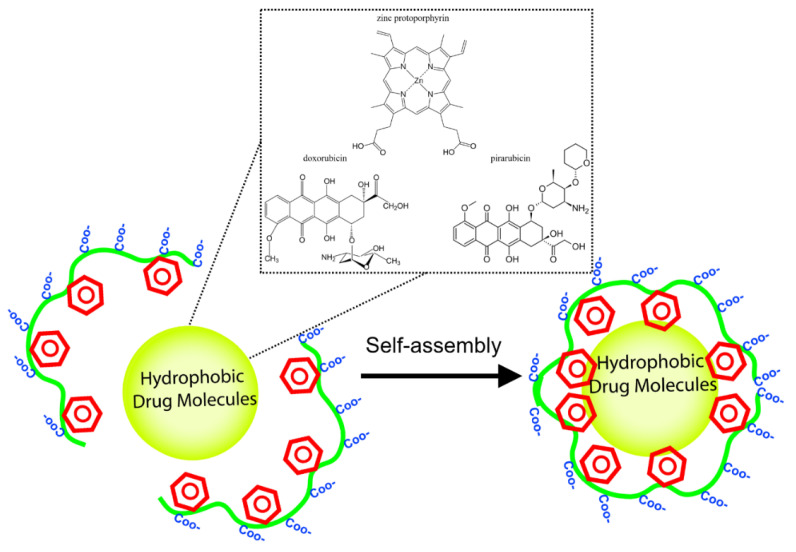
Styrene-maleic acid copolymers were efficient vehicles for encapsulation of hydrophobic drug-like molecules such as doxorubicin, zinc protoporphyrin, pirarubicin.

Maleic anhydride is the only monomer that can be modified post-polymerization, and this has expanded the application of SMAn polymers to biomedical sciences. Polymeric drug delivery has benefited from non-covalent interaction of hydrophobic small molecule drug candidates (such as zinc protoporphyrin, doxorubicin, and pirarubicin) to styrene-maleic acid polymers in order to encapsulate these drugs into micellar constructions with styrene moieties and drug molecules in interior and maleic acid pendant chains in exterior (Figure 4) [42,43,44,45]. The segmental reorientation of styrene and polyanionic maleic acids in SMA copolymer is crucial for its adsorption to hydrophobic ligands while the whole particle remains soluble in aqueous. Such formulations could improve the ultimate bioavailability and bio-efficacy of conjugated drug candidates and decrease their gastrointestinal toxicity. The SMA micellar platform has also transformed the field of membrane structural biology [46].

**Figure 5:**
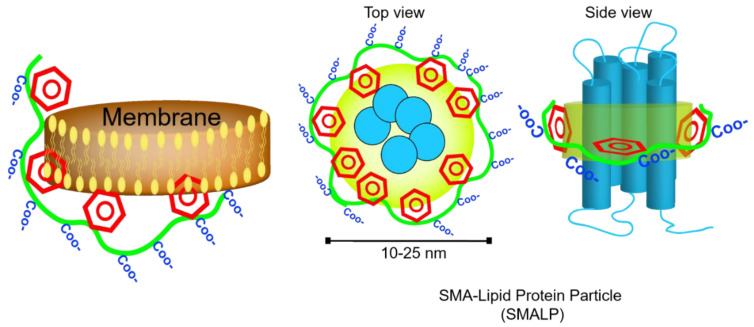
Adsorption, and hydrophobic interaction of styrene moieties (or their hydrophophobic counterparts in other membrane solubilizing polymers such as DIBMA) with acyl chains of phospholipids in lipid bilayer leads to fragmentation the membrane and formation of nanoscale entities called polymer-lipid particles (i.e., SMALP, DIBMALP, etc.).

**Figure 6:**
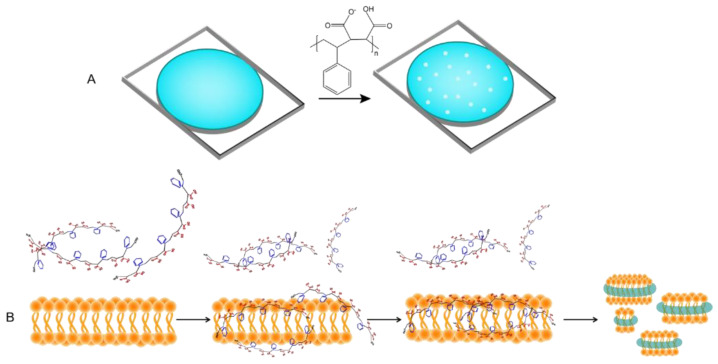
(**A**) SMA polymers form pores in supported lipid bilayer (shown in blue). The same process has been observed in the cells, making them permeable to water and small water-soluble fluorescent molecules (**B**) Schematic view of how encapsulation event perturbs the membrane and leads to membrane fragmentation and formation of discs.

In the absence of hydrophobic ligands, amphipathic SMA copolymer displays hyper coiling behavior so that styrene groups engaged in water-insoluble core and carboxylic acids stay on the surface. This increases the viscosity of solution in salt and pH-dependent manner [43,47]. Notably, the flexibility of the backbone bonds determines the orientation of styrene groups and favors the hydrophobic interactions. The dynamic secondary structures in SMA polymer result in formation of two hydrophobic and hydrophilic active surfaces (which highly resemble amphipathic helices of Apo-I proteins) that can associate with lipid films and form nanometer-sized doughnut-shape particles dubbed Lipodisq [48]. Small-beta barrel protein, PagP, and bacteriorhodopsin (bR) were the first proteins reconstituted and characterized in nanodiscs of DMPC lipids and SMA polymers (also termed SMALP particles) with a diameter of 10–20 nm [49] (Figure 5). Until recently, the specific biophysical behavior of SMA polymers in the interface of membranes was not fully understood. In silico approaches have already been utilized to simulate the behavior of dendrimers [50], polymer-mediated fusion, micelle-lipid interfaces [51], and lipoprotein complexes [52]; therefore molecular dynamic (MD) simulations have proved to be useful to shed light on molecular-scale interaction of SMA and solubilization of biomembrane [53,54]. Coarse-grained (CG) field molecular dynamics simulations and experimental data confirmed the self-aggregation of polyanionic SMA copolymers in solution resulting in globular aggregates [55]. Due to considerable affinity of polymer molecules to membrane (DDPC lipid molecules) that is driven by primary interaction of styrenes with hydrophobic acyl chains, SMA polymers spontaneously (within 20 msec of simulation) and cooperatively insert into adjacent lipid bilayer, bend the membrane at the site of adsorption, then slowly penetrate to the lipid bilayer and localize in the acyl chains of lipids apart from phosphate headgroups, hence leaving styrene groups in perpendicular orientation to lipid acyl chains. While surrounding the lipid bilayer, interestingly, SMA polymer is more stretched (showing higher gyration radius) than free polymers in solution [50]. On the other hand, the encapsulation event perturbs the membrane curvature (by forming a bulge) and planarity, and allows formation of toroidal pores [56] so that water and water-soluble molecules such as fluorescein can permeate inside (Figure 6A) Intriguingly, after encapsulation, the distribution of Na+ ions undergo remarkable changes, as well, and that compensates the repulsion between anionic carboxyl moieties in the lipid-water interface. Small angle X-ray scattering (SAXS) analysis unraveled the details of membrane (composed of either DMPC or POPC) fractionation pathways by SMA 3:1 [55] in which, two−four times more SMA(3:1) is required for lipids in the liquid crystalline phase than in the gel phase to get the lipid vesicles to form lipid-SMA nanodiscs. Under this condition, mixed lipid/SMA(3:1) vesicles coexist with nanodiscs. Above the saturation point, excessive SMA molecules form a belt around the lipid fractions. Of note, temperature, lipid type, and type of SMA polymers play critical roles (Figure 6B).

Furthermore, MD models suggest that relative abundance and the sequence of maleic acid and styrene moieties in the polymer chains may slightly change the behavior of polymers in interaction with model DMPC lipid membrane. For instance, polymers with 2:1 ratio of styrene (S) to maleic acid (MA) completely disaggregate once they integrate with bilayer, whereas SMA polymer with 3:1 ratio of S:MA (comprising a highly ordered sequence of SSS-MA) show higher number of adsorption sites with membrane and maintain their tangled configuration upon insertion into lipid bilayer. Polymers’ net charge (one charge per MA), length (≥1.4 kDa), and sequential polydispersity (SSS ≥ 3) are among the crucial factors that influence the formation and stability of nanodiscs [54].

A relatively universal protocol has been established for SMA-based purification of membrane proteins that are mainly overexpressed in various host organisms and often contain different purification tags [57]. This procedure involves the isolation of membranes and their incubation with SMA polymers in neutral (preferably alkaline) buffers supplemented with glycerol (5–10 *v*/*v*%). Depending on the biophysical and biochemical properties of target proteins, experimental conditions such as temperature, pH, ionic strength, concentration (and type) of SMA polymer and the purification tag are recommended to be finely optimized. These factors, collectively, influence polymer-polymer, polymer-lipid, protein-polymer interactions, which may compromise the yield, purity, activity of final purified target protein, and thus the downstream analyses. Not to mention that different polymers have shown to display differential preference for solubilization of specific types of lipids in biomembranes derived from prokaryotes and eukaryotes [58]. As demonstrated by multiple lines of research, the lipid composition of polymer-based nanodiscs is inevitably susceptible to change through inter-particle collision. Variations in ionic strength, the mass ratio of lipid to polymer and the type of amphipathic polymers, together, control the collision rate and may limit the kinetics of lipid exchange.

Experimental data shows that SMA2:1 (SMA2000) and 3:1 (SMA3000, SZ25010) with an average molecular mass of 7.5–10 kDa can be equally effective in direct purification of membrane proteins from bacterial membrane and spinach chloroplast thylakoids [59,60]. On the contrary, SMA 1:1 (SMA1000), SMA2021, SMA10235, SMA17352, and SZ09008, SZ09006, SZ40005, SZ42010, SZ33030, SZ28065, SZ28110, and SZ2625 were not as useful. Unexpectedly, SMA1440 (1.4:1) displays a remarkable potential for the solubilization of the thylakoid membrane. This perhaps challenges the images that MD simulation models present and call for more pragmatic approaches to opt for the most proper choice of SMA for each target membrane protein.

Despite all the advantages that SMALP technology has offered, there are paramount drawbacks that hinder the utility of this technique for the full spectrum of membrane proteins.

Ionic strength and pH (external factors) and abundance of carboxyl groups of MA monomers (pk_a1_~4 and pK_a2_~9) regulate the overall charge of SMA polymers. These factors are detrimental to formation of secondary structures along the polymer chain and, therefore, to polymer solubility in solution and solubilization of lipid membrane by SMA [61]. In line with this, polyvalent cations (such as magnesium and calcium) and acidic pH compromise the solubility of SMA and limit the utilization of SMA polymers for purification of metal-dependent membrane proteins (e.g., ATP-binding cassette transporter and ATPases) and those which required acidic pH (≤6) for their optimal function (e.g., KcsA potassium channel and lysosomal membrane proteins) [62]. On the other hand, since hydrophobic interactions and self-assembling processes are the driving force for the formation of SMALP nanoparticles, it is not surprising that poly styrene patches of polymers (i.e., higher S:MA ratio) can non-specifically adsorb to hydrophobic patches of proteins (instead of acyl chains of lipids). Likewise, electrostatic interactions with positively charged patches of proteins and polyanionic SMA polymers could negatively impact the folding and function of target proteins. Reportedly, improving batch polydispersity and sequential randomness of SMA polymer could enhance the yield of purification and facilitate the downstream application of isolated nanodiscs via high-resolution techniques such as cryo-EM. Not to mention that aromatic phenyl groups of styrene interfere with far-ultraviolet (UV) spectroscopic analysis of membrane proteins. As such, this approach has undergone many developments to fully optimize the chemical formulation of SMA.

### 3.3. Derivatization of Amphipathic Polymers

Most of the chemical variations in styrene-maleic anhydride polymers came possible through maleic anhydride residues that offer an excellent nucleophilic center. A list of chemicals can be used to convert MAn moieties to their maleamic acid and/or maleimide forms post-functionalization. This approach will reduce the number of carboxylic acids and hence may shift the pK_a_ of polymer macromolecules. Chemicals like Ethanolamine [63], quaternary amines [64], tertiary amines[65], and diamines (e.g., diamino ethyl) [66] have be used for this purpose. Dehydration reaction, on the other hand, adds even more opportunities to increase the variation of active SMA polymers. It is worth mentioning that none of these reactions should neither compromise the solubility of resulting SMA polymers nor slow down their ability to solubilize lipid bilayer. The final products of each synthesis reaction should be verified by analytical methods such as ^13^C NMR, FT-IR, mass spectrometry (MS), and the ability to form nanodiscs of lipid-proteins, as well as size distribution of these particles, should be examined by transmission electron microscopy, light scattering (dynamic light scattering (DLS), static light scattering (SLS)). Generally, the new variants of SMA tend to form larger particles and, due to low abundance of carboxylic groups, have a wider range of pH tolerance and lower sensitively to divalent cations. However, regardless of the type of polymerization reaction utilized for synthesis of parent polymer and the nature of sidechains, the backbone to which such modifications are applied makes a dramatic difference in the results. For instance, use of low molecular weight parent SMAn polymers, 1.6 kDa random copolymer, and 1.3 kDa RAFT (reversible addition-fragmentation chain transfer) polymer), invariably leads to the most optimal products. Unlike conventional random polymerization, RAFT polymerization results in narrower molecular weight distribution, more control over polymer composition and architecture [67]. Small polymers possibly function more like detergents [62,64]. Para and meta Methyl stilbene-MA (STMA) [68] and styrene-acrylic acid (AASTY) copolymers [69] are aromatic polymers that recently introduced as superior alternatives to SMA. These polymers contain 1 to 1 ratio of comonomers, and they feature exemplary alternation in co-monomer sequence (i.e., sequential polydispersity) along polymer chains and batch polydispersity. 

Further, some chemical modifications could expand the application of nanodiscs for drug discovery; one intriguing example is SMA-SH, which is originated from the reactivity of cysteamine with maleic anhydride groups of SMA2000, and contains free thiol groups that can consequently receive thiol-reactive fluorescent probes such as A487 and Atto647N [70]. Some of the sidechain modifications are truly inspirations of natural phospholipid headgroups, zSMA contains zwitterionic phosphatidylcholine (PC) groups grafted to low molecular weight RAFT-polymerized SMAn [62]. The undesired nonspecific interaction between styrenes and protein targets as well as its interference with spectrophotometric techniques (CD, UV-vis, fluorescence) could be alleviated mainly by some chemical modifications, yet this process is so challenging that in many cases, one chooses to build a new polymer by starting a new polymerization reaction using modified styrene residues.

In some cases, non-aromatic (aliphatic) amphipathic polymers have shown to be remarkably effective alternatives for aromatic counterparts. Poly diIsoButylene-*alt*-maleic Acid (DIBMA/Sokalan^®^ CP9 from BASF, Germany) with negative net charge [71] and polymethacrylate (PMA) with positive net charge [72] random copolymer are, respectively, formed by polymerization of diIsobutylene and maleic anhydride comonomers, and butyl methacrylate and cationic methacroylcholine chloride comonomers. DIBMA and PMA can solubilize lipid membranes, and are resistant to changes in pH, perhaps with similar mechanism as SMA. These two polymers are reasonably tolerant to higher concentration of Ca^+2^ cations. DIBMA interacts with acyl chains of phospholipids through the alkyl sidechains, and show minor effect on lipid packing order, and as demonstrated, DIBMA-based nanodiscs (DIBMALPs) show the least collisional lipid transfer [73]. The major pitfall with non-aromatic amphiphilic polymers might involves the significantly low yield of purification of membrane proteins directly extracted from native membranes.

In summary, the current literature suggests the superior popularity of SMA (particularly SMA2:1 and SMA3:1) in addressing fundamental biological questions. The SMALP/DIBMALP-based purification preserves native lipid molecules that surround membrane proteins, offering tremendous opportunity to not only study the lipid composition around a target membrane protein yet also addressing the pivotal roles of lipids in conformational and functional cycles via biophysical tools such as high-resolution X-ray crystallography (especially lipid cubic phase), cryo-electron microscopy (EM), and low-resolution SAXS and SANS, Electron paramagnetic resonance spectroscopy (EPR), and FRET. Previous reports convey considerable underlying efforts to obtain atomic-resolution structures of membrane proteins in nanodiscs for the rational design of novel therapeutics, for instance G-protein-coupled receptors (GPCR) family [74], which account for over 30% of human proteome (some involve in lipid transport) and represent the most challenging drug discovery targets [75]. Some promising examples of such efforts are as follows:

Cryo-electron microscopy of membrane protein-lipid complexes in SMALP nanodiscs displays a state-of-the-art imaging technology for observing the natural lipid bilayer around and buried inside the protein core. Further, the electron density of regulatory and structural lipids can be detected and modeled into the high-resolution EM map of the structure.

**Figure 7:**
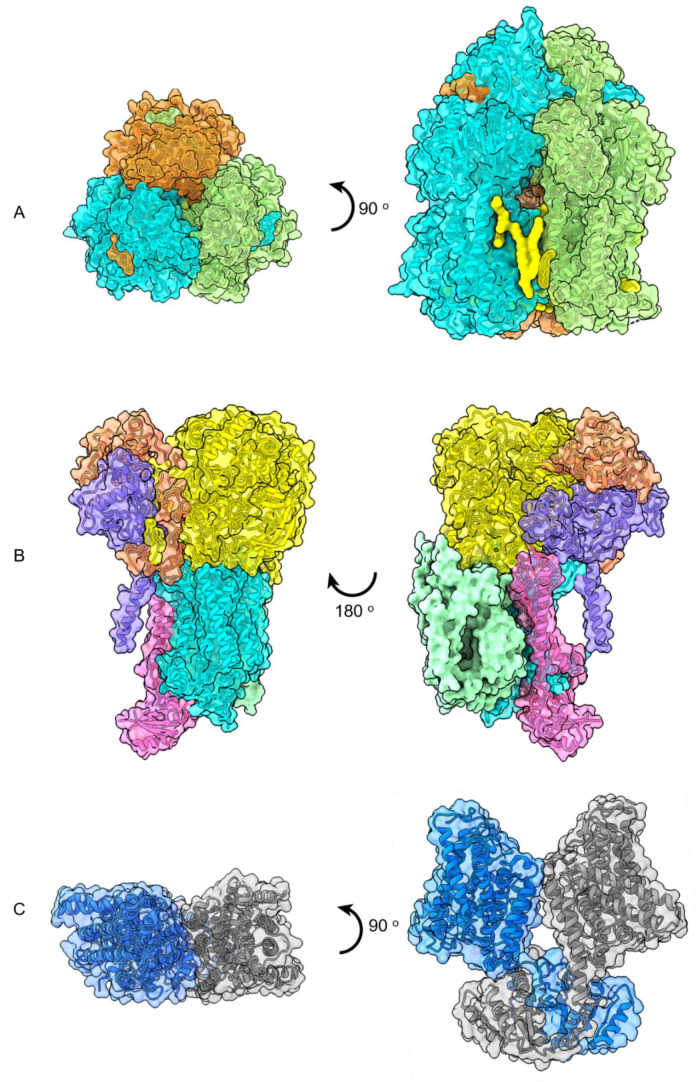

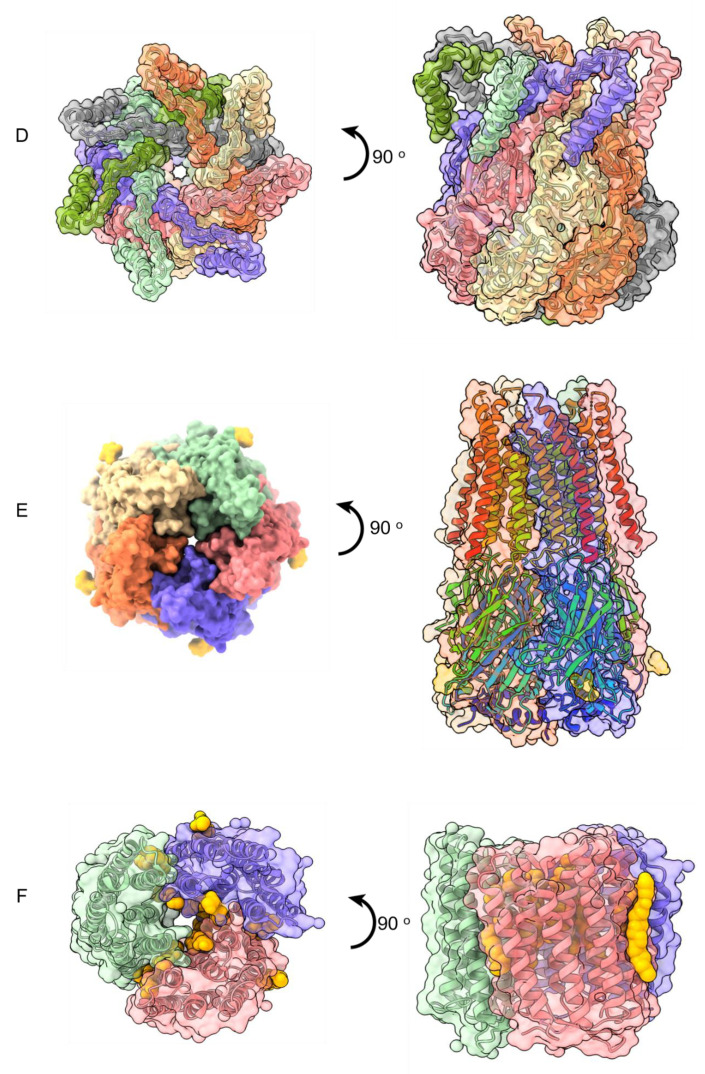

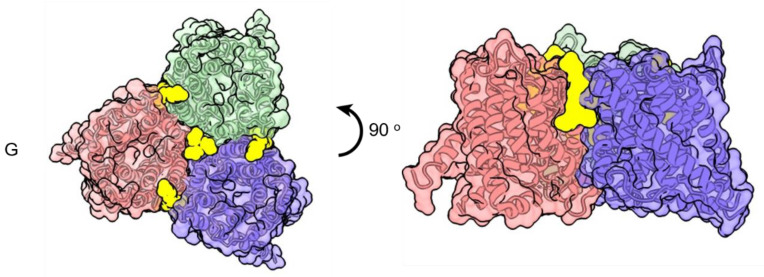
Top and side views of high-resolution cryo-EM structures of (**A**) trimeric AcrB with lipid molecules in yellow (PDB 6BAJ, EMD 7074), (**B**) side views of ACIII photosystem complex (EMD 7286 and 7448), (**C**) dimeric KimA (PDB 6S3K), (**D**) heptameric Ynal (PDB 6RLD, EMD 4990), (**E**) pentameric GlyR (PDB 6PLZ), (**F**) symmetric trimer of Plant *Bd*SLAC1 (PDB 7EN0; EMD-31197). (**G**) The in meso crystal structure of trimeric rhodopsin bound to monoolein after solubilization with SMA. The structure contains three subunits of the trimer (green, blue, red) and nine monoolein lipids (yellow) within the interfaces (PDB 5ITC).

Alternative complex III (ACIII) involves a multi-subunit complex of membrane proteins and plays critical roles in the respiratory and photosynthetic chains of many bacteria. Despite functional analogy with their counterpart cytochrome *bc1* complex, the ACIII and bc1 complexes do not share any structural similarity. The crystal structure of bc1 complex (from bovine heart mitochondria) was first resolved in the 1990s at 2.3 Å resolution in detergent micelles and depleted from their natural lipid molecules [76]. The Gennise lab in 2018 purified and resolved the structure of a functionally active ACIII complex bound to cytochrome c from Flavobacterium Johnsonian [77]. The complex contains all 10 subunits (ActA, ActB, ActC, ActD, ActE, and ActF) and associated cofactors (i.e., [3Fe–4S] cluster, a [4Fe–4S] cluster and six haem c units) in SMALP nanodisc made from SZ25010 and SZ30010 polymers. The EM map shows an unprecedentedly interesting arrangement of subunits. For instance, two subunits bind to lipid bilayer through post-translational modification (N-terminal triacylation of cysteine residues). Also, it displays a thin density of lipid and SMA polymer around a complex. This density was further used to model phosphatidyl ethanolamine (PE) lipid molecules to the structure. The catalytic cycle of the complex is attributed to a core assembly of ActC and ActB that is involved in oxidation of quinol, a haem c assembly consisting of ActA and ActE that directs electrons from ActB to the terminal electron acceptor. The role of transmembrane ActD and ActF subunits remains to be unveiled (Figure 7B).

Another example of a high-resolution image of a multimeric membrane protein embedded within lipid molecules comes from SMA2000-solubilized multidrug efflux protein, AcrB, which contains a hydrophobic core that binds to dyes, and lipophilic antibiotics and even commercial detergents. AcrB protects Gram-negative bacteria against these hazards and therefore causes antimicrobial resistance [78]. AcrB has been an attractive target for biochemists since 2002 when its first asymmetric trimer structure was resolved in n-Dodecyl-B-D-Maltoside (DDM) micelles [79,80,81]. SMA- solubilized AcrB particles have a diameter of 12 nm, of which 9 nm accounts for the width of trimeric protein itself. The cryo-EM density map shows that central cavity of trimer is occupied with 21 low-density lipid molecules (packed in a two layer-triangle) as well as seven annular less-ordered “belt” lipids representing the upper and lower leaflets of bilayer (Figure 7A). Interestingly, due to 3.2 Å resolution of the structure, the thickness of lipid phase (the *Z* coordinates of phosphate headgroups) and the contact points (through hydrogen bonds) between lipid headgroups and amino acids of each subunit (for instance sidechain of arginine and backbone nitrogen of glycine) were quite distinguishable, and that revealed the strikingly asymmetric nature of these interactions, which, in turn, can be attributed to regulatory role of lipids in functional cycles of AcrB. Notably, the architecture and orientation of lipids toward periplasmic (outer leaflet) and cytosolic face appear differently, i.e., outer leaflet lipids shape a loosely packed with curved-shape alkyl chain while those in the inner surface are straight and relatively densely packed. This observation could imply the regulatory role of lipids in conformational changes associated with trimer to keep the central hydrophobic pore in open or closed states. Such high-resolution images of the intimate interaction between a membrane protein and lipid bilayer were also reported in 2005 for two-dimensional (2D) crystals of aquaporin in DMPC synthetic lipids [82,83].

Another millstone in high-resolution structural determination of mammalian membrane proteins using SMA2000-based “native discs” is KimA (previously known as a potassium channel) from Bacillus subtilis in 3.7 Ǻ model with inward-facing occluded homodimers (Figure 7C) (PDB 6S3K, EMD 10092). In vivo and in vitro (in proteolipososmes) transport assays confirm that a proton gradient is required for K+ transport, and H+ transport is K+ dependent, which suggest that KimA is in fact a proton/K+ symporter.

The functional cycles of Glycine receptor (GlyR), a member of Cys-loop or pentameric ligand-gated channel (pLGIC) neurotransmitter receptors, has been purified in the presence of agonist and partial agonists taurine and [84] γ-amino butyric acid GABA [85]. Previous structural data mainly involved full agonist, antagonist, and modulator in detergent-purified truncated forms of GlyR which lacked the M3/M4 cytoplasmic loop. Following SMA-based purification of full-length GlyR and further reconstitution in brain lipids, to elucidate the conformational landscape of GlyR especially pre-open states which were previously uncaptured conformational states along the receptor reaction pathway (Figure 7E). A very recent 2.97-Å resolution cryo-EM model of trimeric plant ion channel *Bd*SLAC1 and its post-translational modification (phosphorylation) dependent-activation profile were characterized in relatively more stable SMALP particles and identified size lipid densities (three central and three peripheral) from outer leaflet for each protomer (with anindependent pore) that is composed of 10 transmembrane helices arranged in five pairs of helical hairpins while TM1, 3, 5, 7, and 9 form the central channel pore with ~6 Å diameter [86]. Due to the flexibility of N- and C-terminal segments, these regions are not included in the current atomic model (EMD-31197).

DIBMA polymers (from either random polymerization or RAFT synthesis) have also been promising in resolving capturing the dynamics of high value protein targets such as MscS-like ion channels notably mechanosensitive ion channel Ynal in an open-like and close-like conformations (PDB 6RLD, EMD 4990) (Figure 7D), in which the backbone of two missing N-terminal transmembrane helices (TM-N1 and TM-N2) could be observed (modeled) [87].

Using lipid cubic phase crystallography, the crystal structure of SMA—solubilized bacteriorhodopsin was resolved at 3.2 Ǻ resolution in synthetic monoolein lipids (Figure 7F) [88,89]. One significant advantage of lipid cubic phase (LCP) is the fact that it does not require highly pure protein samples. Besides, neutral synthetic lipids such as monoacylglycerol (mesophase) substitute the complex natural lipids during reconstitution steps. Although in meso crystallography is not specifically designed for membrane proteins, it provides snapshots of target membrane protein in the synthetic lipid bilayer [90,91].

Natural and synthetic copolymers have been extensively popular in the field of membrane proteins initially in designing optimal detergents and recently used for designing the concept of nanodiscoidal bilayers. As new developments in this field continues to emerge, the need for The SMALP platform enables us to obtain equally informative snapshots from surroundings of membrane proteins; however, it requires more improvement to a need for engineering the amphipathic polymers for formation of larger nanodiscs. Other analytical approaches such as EPR [92], Fluorescence-based methods (FRET/BRET) [93], surface plasmon resonance (SPR) [94], SAXS, and NMR have been utilized to shed light on lipid dependent protein-protein interaction in the lipid bilayer, receptor oligomerizations/regulation and lipid-dependent oligomerization of essential peripheral membrane proteins (such as α-synuclein and Amyloid precursor protein (APP) peptides) [72,88], in polymer-based nanodiscs. The size and lipid composition of nanodiscs are well-controlled during the preparation of LUV vesicles. Moreover, both protein or lipid molecules can be labeled either before or after encapsulation into nanodiscs using antibodies or proper synthetic labels. Since nonspecific interaction between SMA polymers and proteins remains a primary concern, the full activity of post-assembled nanodiscs must be confirmed.

In principle, polymer-based nanodiscs have a broad range of applicability for virtually any biomedically-relevant membrane protein targets in human physiology and pathology.
